# Sulforaphane in cancer precision medicine: from biosynthetic origins to multiscale mechanisms and clinical translation

**DOI:** 10.3389/fimmu.2025.1702860

**Published:** 2025-10-30

**Authors:** Zhipeng Zhao, Qianyue Chen, Xinyu Qiao, Jianjiang Wang, Zaid Tala Abdulqader Ali, Jun Li, Ling Yin

**Affiliations:** ^1^ Department of Rehabilitation Medicine,School of Medicine, Taizhou University, Taizhou, Zhejiang, China; ^2^ Taizhou Municipal Hospital (Taizhou University Affiliated Municipal Hospital), School of Medicine, Taizhou University, Taizhou, Zhejiang, China; ^3^ Institute of Pediatric Neuropsychiatric Diseases, Taizhou, Zhejiang, China; ^4^ The Third School of Clinical Medicine of Zhejiang Chinese Medical University, Hangzhou, Zhejiang, China; ^5^ Beicheng Community Health Service Center, Taizhou, Zhejiang, China; ^6^ Faculty of Medicine, University of Saba Region, Marib, Yemen; ^7^ State Key Laboratory of Oncogenes and Related Genes, Shanghai Cancer Institute, Ren Ji Hospital, School of Medicine, Shanghai Jiao Tong University, Shanghai, China; ^8^ Weill Cornell Medicine, Cornell University, New York, NY, United States; ^9^ College of Medicine, Nanjing Medical University, Nanjing, Jiangsu, China

**Keywords:** glucoraphanin, sulforaphane, Nrf2 signaling, ferroptosis, tumor microenvironment

## Abstract

Sulforaphane (SFN), an isothiocyanate derived from glucoraphanin in cruciferous vegetables, has evolved from a dietary antioxidant to a sophisticated multi-target agent in oncology. While its roles in nuclear factor erythroid 2-related factor 2 (Nrf2) activation and histone deacetylase (HDAC) inhibition are well-established, this review provides a novel synthesis by integrating disparate research scales—a multiscale perspective that spans from the genetic and epigenetic regulation of glucoraphanin biosynthesis in plants to SFN’s recently elucidated effects on ferroptosis, cancer stem cells (CSCs), and the tumor immune microenvironment in humans. We critically evaluate how key host factors, such as gut microbiota composition and glutathione S-transferase (GST) polymorphisms, dictate SFN bioavailability and efficacy, thereby framing a precision nutrition paradigm for its application. Furthermore, we move beyond generic claims of synergy to detail SFN’s specific mechanisms in enhancing conventional therapies, including the modulation of drug transporters and immune checkpoints. By integrating advances from plant biochemistry to molecular oncology, this review establishes an updated and mechanism-oriented framework for realizing SFN’s compelling potential in cancer prevention and therapy through a precision medicine approach.

## 1 Introduction

The growing global burden of cancer necessitates innovative strategies that span prevention, treatment sensitization, and mitigation of therapy-related toxicity (1). In this context, the concept of precision chemoprevention has emerged as a pivotal approach, seeking to leverage individual molecular, genetic, and microbial profiles to tailor interventions for maximum efficacy and minimal risk (2). This paradigm shift is exemplified not only by sulforaphane (SFN) but also by broader research on dietary phytochemicals. For instance, the structural and functional parallels among flavonoids—where subtle chemical differences dictate distinct bioavailability and cancer-modulating activities—highlight a fundamental principle in nutritional oncology: that the efficacy of plant-derived compounds is profoundly influenced by their chemical structure and host-specific factors (3).

Epidemiological studies have consistently linked the consumption of glucoraphanin-rich cruciferous vegetables with a reduced risk of several cancers, including those of the prostate, lung, and colorectum (4). For decades, the mechanistic explanation for this protection has been anchored in two canonical pathways: the activation of the Nrf2-mediated antioxidant response and the inhibition of histone deacetylases (HDACs) (5, 6). However, the scientific narrative of SFN is rapidly expanding. Emerging high-impact research has begun to delineate its capacity to induce iron-dependent ferroptosis, selectively target therapy-resistant CSCs, and remodel the tumor immune landscape, actions that extend far beyond its classical antioxidant and epigenetic roles (7, 8).

The efficacy of SFN is intrinsically linked to the biosynthesis of its precursor, glucoraphanin, in the plant itself. This process is governed by a conserved and finely regulated enzymatic pathway involving Branched-Chain Aminotransferase 4 (BCAT4), Methylthioalkylmalate Synthase 1 (MAM1), and Cytochrome P450 Monooxygenase CYP79F1, with master transcriptional regulators like MYB28 orchestrating the overall flux (9–12). A deep understanding of this biosynthetic machinery is not merely an academic exercise; it provides the foundational knowledge for biofortification strategies, enabling the development of cruciferous crops with enhanced chemopreventive potential.

Despite this expanding mechanistic understanding, a synthesis that adequately captures the full scope of SFN’s journey and action is conspicuously absent. Many are confined to a re-discussion of Nrf2 and HDAC inhibition, lacking integration with its biosynthetic origins and failing to synthesize the rapidly expanding body of evidence on novel and underappreciated mechanisms. Key emerging areas such as the induction of ferroptosis, the selective targeting of CSCs, and the modulation of the tumor immune microenvironment are often omitted or underdeveloped in existing literature. Furthermore, a critical appraisal of the strength of evidence across different experimental models (*in vitro*, *in vivo*, clinical) is frequently absent.

This review is therefore structured to provide a novel and unifying perspective. We first establish the foundation by exploring the bioengineering of the glucoraphanin supply chain. We then trace SFN’s pharmacokinetic journey in the human body, emphasizing the critical roles of the gut microbiome and host genetics. The core of our discussion presents a deep dive into an expanded mechanistic tapestry, where we integrate classical pathways with cutting-edge discoveries in epigenetics (e.g., protein arginine methyltransferase 5 (PRMT5) inhibition), cell death (ferroptosis), and immunomodulation. We quantitatively frame its hormetic behavior (a biphasic dose-response phenomenon characterized by low-dose stimulation and high-dose inhibition) and mechanistically explain its synergistic potential with conventional therapies. Finally, we critically re-evaluate clinical evidence and propose an integrated future direction, arguing that the full potential of SFN will be realized only through a precision medicine approach that accounts for the complex interplay from farm to fork, and from fork to physiology.

## 2 The biosynthetic pathway of glucoraphanin: from gene to metabolite

**Figure 1:**
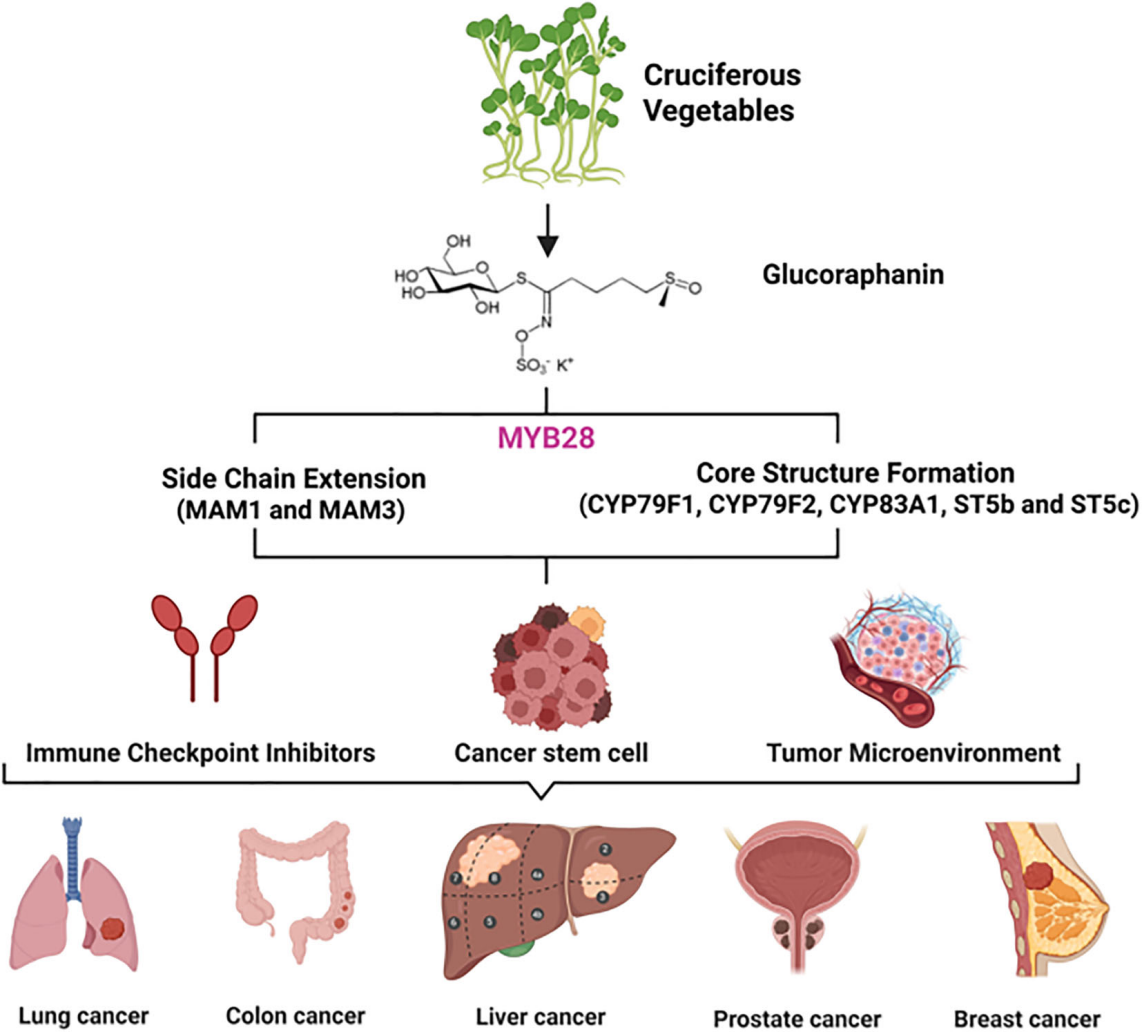
Translational research schematic from natural products in cruciferous vegetables to cancer immunotherapy. As a precursor to the potent anti-cancer and immunomodulatory compound sulforaphane, glucoraphanin provides a molecular foundation and inspiration for developing immune checkpoint inhibitors, showing promise for application in various cancers, including lung, colon, liver, prostate, and breast cancer.

The chemopreventive promise of SFN is fundamentally rooted in the metabolic capacity of its plant source. The biosynthesis of its precursor, glucoraphanin, is a paradigm of specialized metabolism, orchestrated by a conserved pathway that transforms the primary amino acid L-methionine into a potent defense compound (**Figure 1**). Understanding this pathway is not only key to elucidating the origin of SFN but also provides the essential toolkit for its sustainable enhancement through genetic biofortification. This section details the core enzymatic machinery and the multi-layered regulatory networks that govern glucoraphanin accumulation.

### 2.1 The core enzymatic triad: BCAT4, MAM1, and CYP79F1

**Figure 2:**
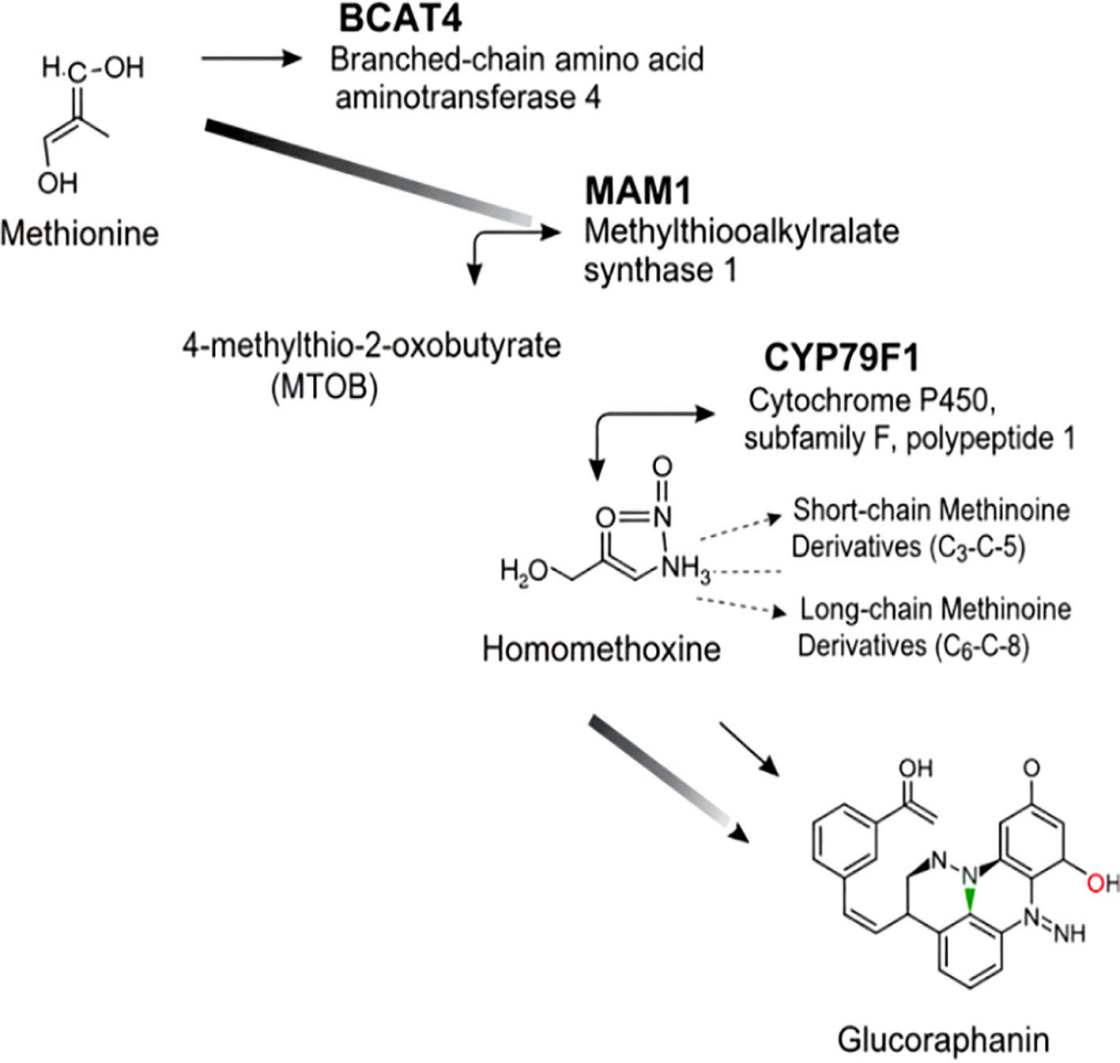
Glucoraphanin biosynthesis pathway. Methionine is first processed by BCAT4 to form MTOB, then MAM1 acts on it. Homomethoxyine and its derivatives (short/long-chain) emerge. Through subsequent steps, glucoraphanin is synthesized, with noted cellular localizations.

The commitment of methionine to aliphatic glucosinolate synthesis is driven by three pivotal enzymes, each executing a distinct and non-redundant step in the construction of the glucoraphanin backbone (**Figure 2**).

#### 2.1.1 BCAT4: The gateway enzyme

Branched-Chain Aminotransferase 4 (BCAT4) initiates the pathway by catalyzing the transamination of L-methionine to 4-methylthio-2-oxobutyrate (MTOB). This reaction serves as the primary and often rate-limiting entry point into the aliphatic glucosinolate system (13). The critical role of BCAT4 in controlling metabolic flux is unequivocally demonstrated by genetic evidence: bcat4 knockout mutants in Arabidopsis thaliana exhibit a dramatic 50–60% reduction in aliphatic glucosinolates and a concurrent 5- to 12-fold accumulation of free methionine (14). The cytosolic localization of BCAT4 implies that its product must be transported into the plastid for subsequent elongation steps, highlighting the sophisticated subcellular compartmentalization of this pathway (15). Furthermore, the inducibility of BCAT4 expression by environmental stresses, such as wounding, illustrates how this primary metabolic enzyme has been co-opted for inducible chemical defense (16).

#### 2.1.2 MAM1: The commitment step in chain elongation

Following initial transamination, Methylthioalkylmalate Synthase 1 (MAM1) catalyzes the first condensation reaction, representing the committing step in side-chain elongation (17, 18). MAM1 exemplifies evolutionary neofunctionalization, having arisen from isopropylmalate synthase (IPMS) through gene duplication. Critical amino acid substitutions remodeled the active site, enabling MAM1 to utilize methionine derivatives, resulting in novel substrate specificity (19). Homology modeling reveals that these mutations create a more expansive substrate-binding pocket, optimally shaped to accommodate short-chain (C3–C5) methionine homologs and distinguishing it from its paralog, MAM3, which specializes in longer-chain (C6–C8) substrates (20). The enzyme also exhibits substrate promiscuity, elongating phenylalanine to produce precursors for 2-phenylethyl glucosinolates (21). Site-directed mutagenesis of key substrate-binding residues in MAM1 alters its product chain length and specificity, demonstrating the enzyme’s plasticity and providing targets for metabolic engineering (22).

#### 2.1.3 CYP79F1: The aldoxime-forming branch point

The pathway converges on Cytochrome P450 Monooxygenase CYP79F1, a critical branch point enzyme that catalyzes the conversion of chain-elongated methionine derivatives (ranging from mono-to hexahomomethionine) to their corresponding aldoximes through N-hydroxylation reactions, representing a critical branch point in aliphatic glucosinolate biosynthesis (23). Biochemical characterization of recombinant CYP79F1 expressed in Escherichia coli confirmed this enzymatic transformation generates highly reactive (E)-and (Z)-aldoxime intermediates essential for subsequent glucosinolate formation (24). The enzyme exhibits distinct substrate preferences with particularly high affinity for di-and trihomomethionine, explaining the predominance of 4C-and 5C-glucosinolates in Arabidopsis (25). Genetic evidence from CYP79F1 knockout mutants confirms its essential role, as these plants completely lack short-chain aliphatic glucosinolates while accumulating methionine-derived precursors (26). Spatial expression patterns predominantly in photosynthetic tissues and reproductive organs correlate with tissue-specific glucosinolate accumulation (27). Notably, CYP79F1 shows partial functional redundancy with its paralog CYP79F2, which specializes in longer-chain (5C-6C) substrates, providing metabolic flexibility in glucosinolate profiles (28).

### 2.2 Transcriptional and epigenetic regulation: a multi-layered control system

**Figure 3:**
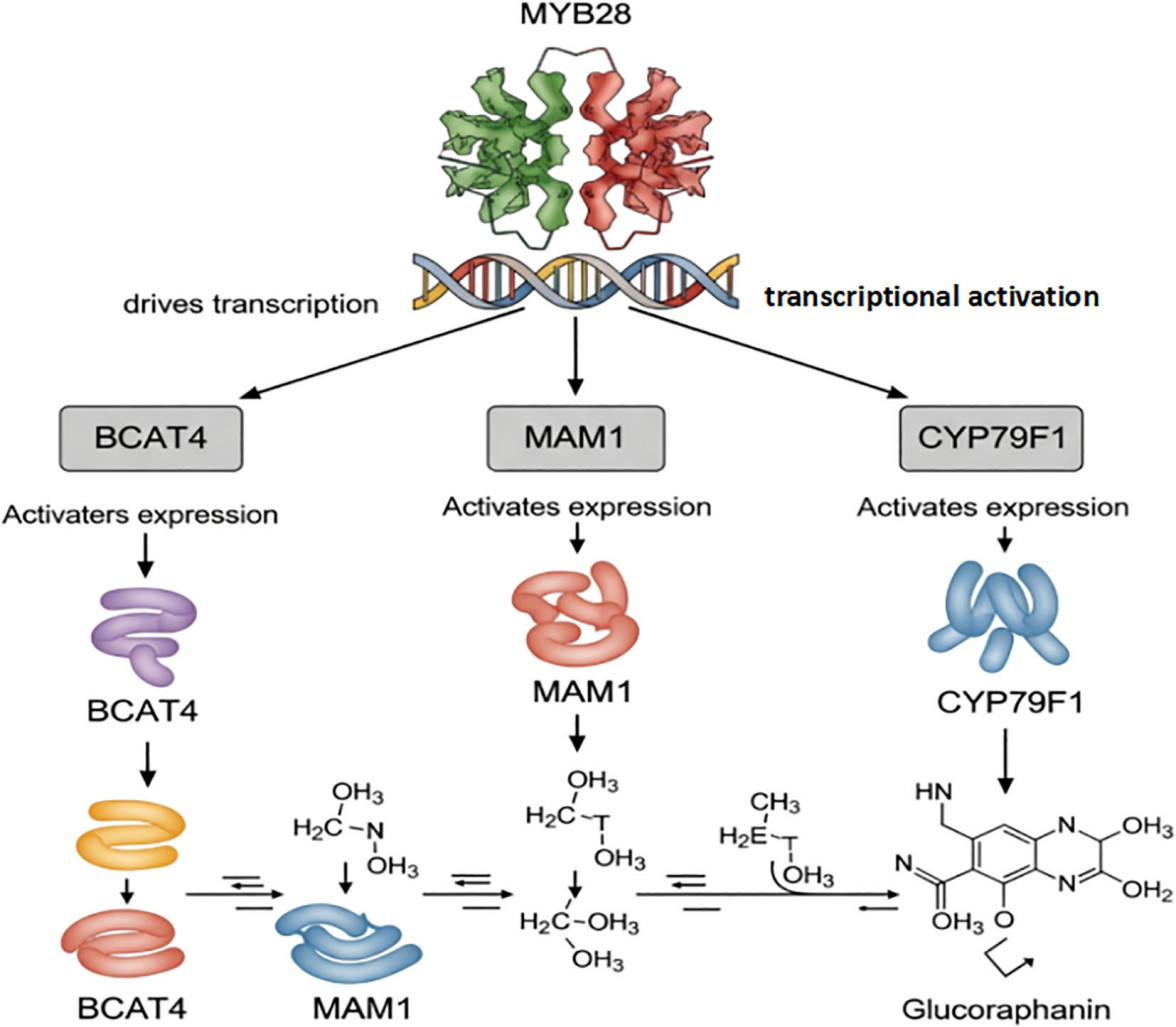
Transcriptional activation of glucoraphanin biosynthetic genes by MYB28. MYB28 directly activates the transcription of key biosynthetic genes (BCAT4, MAM1, and CYP79F1), which encode enzymes that catalyze sequential steps in glucoraphanin synthesis. These enzymes convert precursor molecules into glucoraphanin through enzymatic reactions.

The precise spatial and temporal accumulation of glucoraphanin is dynamically controlled by a sophisticated regulatory regime that integrates transcriptional, epigenetic, and post-translational cues (**Figure 3**).

An R2R3-MYB transcription factor has been identified as a master regulator (29). It activates genes like MAM1, MAM3, CYP79F1, CYP79F2, CYP83A1, which are involved in both side-chain elongation and core structure formation of aliphatic glucosinolates, thereby influencing glucoraphanin accumulation (30). The coordinated regulation of these genes ensures the proper synthesis and accumulation of glucoraphanin (31).

The transcriptional activity of this regulator is tightly modulated by epigenetic modifications in response to sulfur availability (32). ChIP-qPCR analysis revealed that under sulfur deficiency conditions, the ratio of activating H3K4me3 to repressive H3K27me3 marks at its locus is significantly altered, resulting in reduced transcript abundance (33). This chromatin state transition is mediated by the SWR1 chromatin remodeling complex, as evidenced by co-immunoprecipitation experiments showing physical interaction between the ARP6 subunit and the promoter region (34). DNase I hypersensitivity assays further demonstrated that sulfur deficiency increases nucleosome occupancy, preventing transcription factor access (35).

Post-translational regulation occurs primarily through mitogen-activated protein kinase (MAPK)-mediated phosphorylation (36). *In vitro* kinase assays using recombinant MPK6 and protein fragments identified a critical phosphorylation site (37). This modification enhances the interaction with the MED25 mediator subunit, as measured by surface plasmon resonance (38). Transient expression assays in protoplasts confirmed that phospho-mimetic mutants exhibit higher transcriptional activation of target promoters compared to wild-type protein (39).

### 2.3 Pathway integration and compartmentalized activation

Together, BCAT4, MAM1, and CYP79F1 form the core enzymatic triad of the aliphatic glucosinolate biosynthetic pathway, each executing distinct and non-redundant functions in stepwise glucoraphanin construction from amino acid precursors. Their coordinated expression and activity are tightly regulated at transcriptional and post-transcriptional levels, often in response to developmental cues, environmental stimuli, and hormonal signals. In Chinese kale, these genes exhibit tissue-specific and developmental regulation, with activity peaks frequently coinciding with active glucoraphanin accumulation periods (40).

Building upon this core machinery, the glucosinolate pathway involves three additional enzymatic modules completing metabolic transformation: (1) CYP83A1 converts aliphatic aldoximes to thiohydroximates, (2) UGT74B1 mediates glycosylation of sulfated intermediates (requiring prior sulfation by SOT17/18), (3) the myrosinase-TGG1/2 system, in concert with epitope-specific proteins (ESPs), hydrolyzes stored glucosinolates into bioactive compounds like SFN upon tissue damage (41). This sequential transformation from oxime to thiohydroximate through sulfation and glycosylation, culminating in hydrolysis, is governed by strict spatial compartmentalization, with vacuolar-localized myrosinases and apoplastic ESPs coordinating precise temporal regulation of glucosinolate activation (42).

## 3 Interindividual variability and precision response: host factors governing SFN bioavailability and efficacy

The journey of SFN from dietary intake to systemic bioactivity is a complex process governed by a series of metabolic conversions and, crucially, modulated by significant inter-individual variation. Understanding this journey is not merely a pharmacokinetic exercise but is fundamental to explaining the disparate outcomes observed in clinical trials and for designing precision-based interventions. This section moves beyond a deterministic view of SFN bioavailability to focus on the host factors—specifically the gut microbiome and host genetics—that act as key determinants of efficacy.

### 3.1 The metabolic fate of glucoraphanin and SFN

Upon ingestion of cruciferous vegetables, the inert precursor glucoraphanin is hydrolyzed to its bioactive form, SFN, by the enzyme myrosinase. This conversion can be initiated by plant-derived myrosinase, released upon tissue damage (e.g., chewing), or by microbial myrosinase in the gut (43). The resulting SFN is rapidly absorbed in the small intestine and undergoes extensive phase II metabolism in the liver, primarily via the mercapturic acid pathway. This involves sequential conjugation with glutathione (catalyzed by glutathione S-transferases, GSTs), followed by enzymatic processing to yield SFN-cysteine-glycine, SFN-cysteine, and ultimately SFN-N-acetylcysteine (SFN-NAC), which are the primary metabolites detected in plasma and urine (44, 45).

The pharmacokinetic profile of SFN is characterized by rapid absorption, with peak plasma concentrations of SFN and its metabolites occurring within 1–3 hours post-consumption, and a relatively short elimination half-life (46). Despite this rapid clearance, SFN and its conjugates effectively distribute to various tissues, including the prostate, lung, and bladder, where they can accumulate at concentrations sufficient to exert biological effects, as demonstrated in both rodent models and human tissue biopsies (47, 48).

### 3.2 The gut microbiome: a metabolic gatekeeper

A pivotal, and often rate-limiting, step in SFN activation is the hydrolysis of glucoraphanin by the gut microbiota. Individuals harbor vastly different communities and abundances of myrosinase-producing bacteria (e.g., certain strains of Bacteroides, Enterococcus, and Lactobacillus), leading to profound differences in the efficiency of SFN generation (49, 50). This variability explains why the bioavailability of SFN from cooked vegetables (where plant myrosinase is inactivated) can differ dramatically between individuals. The pivotal role of the gut microbiota is underscored by evidence demonstrating that the protective effects of a steamed broccoli sprout diet against colitis are entirely dependent on its presence, as it generates bioactive sulforaphane in the colon even when the plant’s own myrosinase is inactivated (51). This foundational understanding opens the door for novel interventions aimed at modulating the microbial community itself. Consequently, the administration of probiotic supplements is being explored as a strategic “bio-therapy” to standardize and enhance the conversion of glucoraphanin to SFN, thereby ensuring a more reliable and sustained delivery of the bioactive compound from dietary sources (52).

### 3.3 Host genetics: GST polymorphisms and metabolic destiny

Beyond microbial activation, host genetics, particularly polymorphisms in GST genes, play a decisive role in shaping SFN’s metabolic fate and tissue retention. GST enzymes, especially GSTM1 and GSTT1, are responsible for conjugating SFN with glutathione, a step traditionally viewed as a detoxification and excretion pathway.

Additionally, polymorphisms in other genes involved in SFN metabolism and response have been explored. For instance, the Nad(p)h: quinone oxidoreductase (NQO1)*2 polymorphism has been associated with altered efficacy of SFN. Research shows that SFN can restore NQO1 enzyme activity in leukemia cells carrying this polymorphism, which normally results in reduced activity (53). Furthermore, interactions between SFN and Glutathione S-Transferase P1 (GSTP1) gene variants have been observed, where SFN can modulate the expression levels of different GSTP1 haplotypes (54). These findings suggest that a broader genetic profiling beyond GSTM1 and GSTT1 may further refine the precision application of SFN.

Crucially, for the most extensively studied GST polymorphisms, the metabolic impact is profound and counterintuitive. Individuals with null genotypes for GSTM1 or GSTT1 (i.e., they lack functional copies of these genes) exhibit a markedly different pharmacokinetic profile. Contrary to the assumption that faster conjugation diminishes efficacy, these individuals demonstrate significantly higher and more prolonged levels of unconjugated, bioactive SFN in the bloodstream (55, 56). The proposed mechanism is that in the absence of efficient GST-mediated conjugation, SFN is cleared more slowly, allowing it to circulate in its active form for a longer duration and potentially exert stronger biological effects (57).

This genetic stratification has profound clinical implications. It suggests that GST null individuals may be the “optimal responders” to SFN supplementation. Indeed, several chemoprevention trials have reported that the reduction in biomarkers of cancer risk (e.g., aflatoxin-DNA adducts) following SFN intervention was predominantly observed in subjects with the GSTM1-null genotype (58). This evidence necessitates a paradigm shift from a one-size-fits-all supplementation approach to a genotype-stratified strategy, wherein GST status could be used to identify individuals most likely to benefit from SFN-based prevention.

This genotype-dependent efficacy is robustly supported by epidemiological and clinical evidence in colorectal cancer, particularly for individuals with combined GSTM1 and GSTT1 null genotypes. A nested case-control study within the Singapore Chinese Health Study demonstrated a significant 57% reduction in colon cancer risk among high consumers of dietary isothiocyanates (ITCs) who carried the double null genotype, suggesting that compromised GST activity enhances the protective effect of ITCs like SFN (59). This interaction is mechanistically consistent with the observation that the protective effect of high broccoli intake against colorectal adenomas was exclusively evident in individuals with the GSTM1-null genotype, likely due to prolonged tissue exposure to bioactive ITCs (60). Further corroborating this, a UK-based study found that the protective effect of vegetable consumption against colorectal cancer was primarily confined to individuals with a deficient or intermediate GSTT1 phenotype (61). Furthermore, the protective association is significantly modified by age and smoking status. Specifically, the strongest inverse association between cruciferous vegetable intake and colon cancer risk is observed among younger individuals (particularly those under 55) with the GSTM1-null genotype, and the benefit appears more pronounced in smokers (62). This underscores that GSTM1 and GSTT1 genotypes are key determinants for stratifying individuals who would derive maximum benefit from SFN-based chemoprevention for colorectal cancer, with age and smoking history providing critical contextual refinement.

By integrating the roles of the gut microbiome and host genetics, it becomes clear that the biological activity of SFN is not solely a function of the ingested dose. Instead, it is an emergent property of the complex interaction between diet, microbiota, and the host’s genomic landscape. Acknowledging and accounting for these determinants is the cornerstone of translating SFN’s promise into predictable and potent clinical outcomes.

## 4 The multiscale anticancer mechanisms of SFN: beyond Nrf2 and HDAC inhibition

While the activation of Nrf2-mediated antioxidant response and inhibition of HDACs represent well-established mechanisms underlying SFN’s anticancer properties, emerging evidence reveals a far more complex pharmacological profile. This section delineates SFN’s multifaceted mechanisms across epigenetic regulation, programmed cell death pathways (particularly ferroptosis), and tumor immune microenvironment remodeling, establishing its role as a truly multi-targeted therapeutic agent.

### 4.1 Revisiting classical pathways: context-dependent roles of Nrf2 and HDAC inhibition

SFN activates the Nrf2 pathway through covalent modification of specific cysteine residues (Cys151, Cys273, and Cys288) on the Keap1 protein, leading to Nrf2 stabilization, nuclear translocation, and transcriptional activation of cytoprotective genes including NAD(P)H quinone oxidoreductase 1 (NQO1), heme oxygenase-1 (HO-1), and glutathione biosynthesis enzymes (6, 8). However, the role of Nrf2 in cancer demonstrates significant context-dependency. While Nrf2 activation provides chemopreventive benefits in preneoplastic and normal cells, its persistent activation in established tumors may paradoxically promote cancer cell survival and confer resistance to conventional chemotherapy (63). This dual nature underscores the critical importance of precise dosing and timing in SFN-based interventions. Specifically, while chronic, low-dose SFN may be ideal for prevention, its use as an adjunct to chemotherapy in established cancers requires careful scheduling to avoid potential protection of tumor cells.

Similarly, SFN’s function as an HDAC inhibitor extends beyond histone hyperacetylation, establishing it as a broad-spectrum epigenetic modulator in cancer chemoprevention. Its HDAC inhibitory activity directly contributes to the reactivation of silenced tumor suppressor genes and is a key mechanism underlying its remarkable anti-tumor effects in urologic and other cancers, as observed both *in vitro* and *in vivo* without significant toxicity (64). This epigenetic intervention engages in extensive cross-talk, potentially through global demethylation and modulation of microRNA expression, thereby reversing aberrant gene transcription profiles in cancer (65). Furthermore, SFN orchestrates a multi-pronged assault on cancer cells by promoting the acetylation of non-histone proteins such as p53, and synergistically activating critical pathways including cell cycle arrest, apoptosis, and sensitization to other therapeutic agents like TRAIL, which is particularly promising for targeting therapy-resistant cases (66).

### 4.2 Ferroptosis induction: an emerging cell death mechanism

Beyond its established roles in apoptosis and cell cycle arrest, SFN demonstrates significant capacity to induce ferroptosis—an iron-dependent form of regulated cell death characterized by lethal lipid peroxide accumulation. This emerging mechanism substantially expands our understanding of SFN’s anticancer portfolio, particularly against therapy-resistant malignancies. The electrophilic nature of SFN drives its direct conjugation with glutathione, effectively depleting intracellular GSH pools and consequently inhibiting glutathione peroxidase 4 (GPX4) activity. As GPX4 serves as the master regulator of lipid hydroperoxide reduction, its suppression triggers irreversible lipid peroxide accumulation that culminates in ferroptotic cell death (67, 68).

Complementing this primary mechanism, emerging evidence indicates that SFN modulates iron metabolism through upregulation of ferritin heavy chain (FTH1), potentially altering intracellular iron homeostasis to promote iron-mediated lipid peroxidation via Fenton chemistry (69, 70). This coordinated assault on cellular antioxidant defenses and iron regulation proves particularly effective against CSCs, whose elevated basal oxidative stress status renders them exquisitely vulnerable to SFN-induced ferroptosis.

### 4.3 Targeting CSCs through coordinated pathway disruption

SFN demonstrates remarkable efficacy against CSCs through its ability to simultaneously disrupt multiple signaling pathways that maintain stemness and self-renewal capacity. The compound orchestrates a multi-pronged assault on the core regulatory networks that sustain these treatment-resistant cell populations, addressing a fundamental challenge in cancer therapeutics. At the heart of SFN’s anti-CSC activity lies its coordinated interference with Wnt/β-catenin, Notch, and Hedgehog signaling - three evolutionarily conserved pathways that frequently become dysregulated in CSCs. SFN promotes the phosphorylative degradation of β-catenin while concurrently suppressing downstream targets including c-Myc and cyclin D1, effectively dismantling the transcriptional program that drives CSC self-renewal (71). This disruption of Wnt signaling creates a permissive environment for CSC differentiation and loss of tumor-initiating potential.

Complementing this mechanism, SFN demonstrates sophisticated regulation of the Notch pathway through a cascade of molecular events. In lung cancer models, SFN suppresses ΔNp63α expression, which in turn reduces IL-6 secretion and inhibits Notch1 signaling activation. The resulting diminishment of Hes1 expression and other Notch effectors compromises the sphere-forming ability of CSCs and their capacity to maintain the undifferentiated state (72, 73). This multi-layered approach to Notch pathway inhibition represents a particularly effective strategy given the pathway’s crucial role in cell fate decisions.

Emerging evidence further suggests that SFN may interfere with Hedgehog signaling through modulation of Gli transcription factor function, though the precise mechanisms require additional validation (74). The coordinated nature of these pathway disruptions is particularly significant, as CSCs often demonstrate remarkable plasticity and can maintain their stem-like properties through compensatory activation of alternative signaling routes when individual pathways are targeted in isolation.

### 4.4 Remodeling the tumor immune microenvironment

Beyond its direct cytotoxic effects on cancer cells, SFN demonstrates a remarkable capacity to remodel the tumor immune microenvironment through multifaceted immunomodulatory mechanisms. This repositioning of the host immune system against established tumors represents a crucial dimension of SFN’s anticancer activity. Mechanistic studies reveal that SFN significantly downregulates programmed death-ligand 1 (PD-L1) expression on tumor cells through multiple mechanisms, including direct covalent modification of cysteine residues on STAT1, which inhibits its transcriptional activity and blocks IFN-γ-induced PD-L1 expression (75). This checkpoint modulation creates permissive conditions for T cell-mediated tumor elimination and provides strong rationale for combining SFN with immune checkpoint inhibitors to overcome therapeutic resistance.

The immunomodulatory effects of SFN extend to comprehensive reprogramming of immune cell populations within the tumor niche. SFN treatment effectively suppresses the accumulation and immunosuppressive functions of myeloid-derived suppressor cells (MDSCs). This is mechanistically demonstrated in breast cancer models, where SFN, by activating the Nrf2 pathway, reduces the secretion of prostaglandin E2 (PGE2) from tumor cells, thereby triggering MDSCs to switch from an immunosuppressive to an immunogenic phenotype and inhibiting their expansion (76). The immunomodulatory prowess of SFN is further exemplified by its ability to reprogram macrophage polarization and recalibrate T-cell immunity. In an immunocompetent mouse model of hepatitis B virus (HBV) infection, SFN treatment significantly promoted the repolarization of macrophages towards the antitumoral M1 phenotype, as evidenced by increased expression of Cd86 and iNOS, and inhibited the expression of Arg1. Concurrently, SFN altered the adaptive immune balance by increasing the proportion of pro-inflammatory Th17 cells and decreasing the Treg/Th17 ratio. Mechanistically, these immunomodulatory effects were driven by SFN-mediated inhibition of macrophage migration inhibitory factor (MIF). This comprehensive reprogramming of both innate and adaptive immunity underscores SFN’s potent capacity to alleviate immunosuppression and restore effective anti-tumor and anti-viral immunity (77).

Emerging evidence further indicates that SFN promotes the repolarization of tumor-associated macrophages from the protumoral M2 phenotype toward the antitumoral M1 state. This macrophage reprogramming is associated with SFN-mediated inhibition of the transcription factor c-Myc, which normally drives M2 polarization, and concurrent activation of the Nrf2 pathway that favors M1-associated gene expression profiles (78). Through this coordinated regulation of both adaptive and innate immune components, SFN establishes a more immunostimulatory microenvironment that not only enhances direct tumor cell killing but also creates favorable conditions for combination strategies with various immunotherapeutic approaches.

### 4.5 Expanded epigenetic regulation: PRMT5 inhibition

Beyond its established HDAC inhibitory activity, SFN demonstrates additional epigenetic modulation through inhibition of protein arginine methyltransferase 5 (PRMT5). This enzyme catalyzes symmetric dimethylation of histone H3R8 and H4R3, modifications associated with transcriptional repression of tumor suppressor genes. In mesothelioma models, SFN disrupts PRMT5/MEP50 complex function, inhibiting its methyltransferase activity, reactivating tumor suppressor expression, and suppressing cancer cell proliferation, invasion, and stem-like properties (79, 80). This mechanism further establishes SFN as a multi-valent epigenetic modulator with broad therapeutic potential.

This comprehensive analysis of SFN’s multiscale mechanisms provides the necessary foundation for developing targeted therapeutic strategies that maximize its anticancer efficacy while minimizing potential resistance mechanisms. The integration of these diverse pathways underscores SFN’s unique position as a naturally derived agent with sophisticated, multi-modal activity against cancer.

The multifaceted anticancer mechanisms of SFN have been demonstrated across a wide spectrum of malignancies, extending far beyond its classical roles. As summarized in **Table 1**, the compound exerts potent effects—including inhibition of proliferation, induction of apoptosis, suppression of CSCs, and modulation of the tumor immune microenvironment—across diverse cancer types. These pleiotropic actions are mediated through a complex network of interconnected molecular pathways, underscoring SFN’s value as a multi-targeted agent in oncology and providing a mechanistic basis for its synergy with conventional therapies.

**Table 1:** Summary of anticancer effects and underlying mechanisms of SFN.

Cancer type	Effects of SFN	Mechanisms involved	References
Prostate Cancer	Synergizes with paclitaxel to induce apoptosis; inhibits cell growth.	HDAC inhibition; enhanced activation of apoptotic pathways.	(81)
Breast Cancer	Induces cell cycle arrest and apoptosis; inhibits proliferation	Upregulation of CDK5R1; combinatorial epigenetic modulation (e.g., with genistein)	(58, 82)
Gastric Cancer	Suppresses gastric cancer cell growth; impairs the efficacy of immune checkpoint blockade therapy (α-PD-L1 mAb).	Activation of the ΔNp63α/PD-L1 axis.	(83)
Colorectal Cancer	Suppresses cancer stem cell (CSC) properties; inhibits proliferation.	Transcriptional inhibition of Nanog/Oct4/Sox2 expression through downregulation of ΔNp63α.	(84)
Bladder Cancer	Inhibits cancer cell growth.	Induction of phase II enzymes; HDAC inhibition.	(48, 55)
Glioblastoma	Induces apoptosis.	Activates tumor-associated macrophages which induce tumor cell death; Inhibition of the α-tubulin/PD-L1/PFKFB4 axis.	(85)
Melanoma	Reduces ultraviolet-induced skin damage and erythema; modulates protumorigenic cytokines.	Enhanced expression of phase II enzymes; reduced inflammatory responses; systemic immunomodulation.	(86)
Intrahepatic Cholangiocarcinoma	Synergizes with gemcitabine.	HDAC inhibition and restoration of pro-apoptotic gene expression.	(5)
Mesothelioma	Suppresses proliferation, invasion, and stem-like properties.	Inhibition of PRMT5/MEP50 complex function.	(80)
Lung Cancer	Inhibits cell migration, invasion, and metastasis; suppresses the acquisition of CSC-like properties.	Inhibition of epithelial-mesenchymal transition (EMT) via ERK5 activation; Suppression of CSC self-renewal via the IL-6/ΔNp63α/Notch axis.	(72, 73)
Pancreatic Cancer	Inhibits growth and metastasis; induces cell death.	ROS-mediated apoptosis and AMPK/Nrf2 pathway activation.	(87, 88)
Liver Cancer	Induces cell cycle arrest, apoptosis, and DNA damage; suppresses proliferation.	HDAC inhibition; modulation of DNA methylation; downregulation of MAP kinases; upregulation of DNA damage response genes.	(89)
Ovarian Cancer	Induces cell cycle arrest and apoptosis; reverses cisplatin resistance.	G2/M arrest via disruption of Cyclin B1/CDC2 complex; enhanced cisplatin sensitivity via miR-30a-3p-mediated suppression of ERCC1 (DNA repair) and ATP7A (drug efflux).	(90, 91)
Head and Neck Squamous Cell Carcinoma (HNSCC)	Exerts chemopreventive potential; induces robust biomarker expression.	NRF2-dependent upregulation of oxidative stress-responsive genes (e.g., HMOX1, HSPA1A) and NKG2D ligands (MICA/B).	(92)

## 5 Synergistic therapy and clinical translation

The significant interindividual variability in SFN response is not a barrier but an opportunity for precision medicine. A critical synthesis of the literature reveals that the efficacy of SFN is profoundly influenced by host genetics and the gut microbiome. As detailed in the preceding sections, SFN’s bioavailability and effects are significantly modulated by key host factors. To systematically articulate this precision medicine paradigm, **Table 2** summarizes the evidence-based strategies for the individualized application of SFN. This paradigm shift from a one-size-fits-all supplementation strategy is fundamental to realizing the full clinical potential of this dietary phytochemical.

**Table 2:** Strategies and evidence for a precision medicine approach to SFN application.

Predictive factor/strategy	Evidence and application in cancer precision medicine	References
Host Genetics (GST Polymorphisms)	Cancer Types: Prostate Cancer, Bladder Cancer, Colon Cancer • The GSTM1-null genotype is associated with greater accumulation of bioactive SFN in prostate tissue, providing a pharmacokinetic basis for its enhanced efficacy in prostate cancer prevention. This genotype also correlates with a more favorable SFN metabolic profile in bladder cancer, supporting its role as a predictive biomarker. • In colon cancer, the null genotypes of GSTM1 and/or GSTT1 are linked to enhanced SFN accumulation in colonic tissue and greater efficacy in suppressing carcinogen-DNA adducts and inflammatory pathways.	(48, 55, 59–62, 93)
Host Genetics (Other Polymorphisms)	Cancer Type: Leukemia, Lung Cancer • Polymorphisms in genes such as NQO1 and GSTP1 influence response to SFN. SFN can restore NQO1 activity in NQO1*2 polymorphic cells, and modulates expression of specific GSTP1 haplotypes.	(53, 54)
Gut Microbiome Composition	Cancer Type: Hyperuricemia-associated Cancer Risk • SFN reprograms the gut microbiome and metabolome, enhancing microbial diversity and improving metabolic function. This remodeling of the gut environment may reduce the risk of chronic conditions like hyperuricemia that are linked to increased cancer risk.	(49)
Advanced Formulation Strategies	Cancer Type: Breast Cancer • Preclinical studies demonstrate that nanotechnology-based delivery systems enhance SFN stability and enable tumor-specific delivery in breast cancer models, markedly improving its antitumor efficacy and overcoming pharmacokinetic limitations.	(94, 95)
Dietary Source Biofortification	Cancer Type: Broad-Spectrum Prevention (hypothetical) • CRISPR-Cas9-mediated genome editing of Brassica crops has successfully generated varieties with enhanced glucoraphanin content, providing a sustainable strategy for population-level precision chemoprevention.	(9, 96)

### 5.1 Mechanisms of synergy with conventional therapeutics

SFN’s multi-targeted nature makes it an ideal candidate for combination therapy, as it can sensitize cancer cells to conventional treatments through several complementary avenues. SFN demonstrates remarkable capacity to enhance the efficacy of conventional cancer treatments through multiple complementary mechanisms. In combination with chemotherapeutic agents, SFN modulates key cellular pathways that influence drug sensitivity and resistance. The compound significantly enhances the effectiveness of gemcitabine in intrahepatic cholangiocarcinoma by inhibiting HDAC activity and restoring expression of pro-apoptotic genes (5). Similarly, SFN synergizes with paclitaxel in prostate cancer models through amplified activation of apoptotic pathways, demonstrating the potential to reduce required chemotherapeutic doses while maintaining therapeutic efficacy (81).

Beyond direct enhancement of cytotoxic effects, SFN provides protection against therapy-induced damage to normal tissues. The compound’s ability to activate the Nrf2-mediated antioxidant response helps mitigate the collateral damage caused by radiation and chemotherapy, particularly in highly vulnerable tissues such as hematopoietic systems and mucosal barriers (6). This cytoprotective effect, when strategically timed and dosed, could significantly improve patients’ tolerance to aggressive treatment regimens.

### 5.2 Clinical evidence and trial outcomes

The translation of SFN from preclinical models to human clinical applications has generated substantial evidence supporting its potential in cancer prevention and management. Multiple well-designed clinical trials have demonstrated SFN’s biological activity and therapeutic potential, while also revealing important considerations for its clinical implementation (**Figure 3**).

In the realm of cancer prevention, a landmark randomized controlled trial investigated the effects of broccoli sprout beverage in Chinese populations exposed to high levels of air pollution (97). The study demonstrated that SFN supplementation significantly enhanced the excretion of airborne pollutants, including benzene and acrolein, through the mercapturic acid pathway. This finding provides compelling evidence for SFN’s chemopreventive potential in high-risk populations, establishing its role in enhancing detoxification of environmental carcinogens.

For prostate cancer management, clinical evidence has been particularly promising. A phase II clinical trial examined the effects of SFN-rich broccoli sprout extracts in men with recurrent prostate cancer following radical prostatectomy (98). The study revealed that SFN supplementation significantly modulated gene expression profiles in prostate tissue, with upregulation of genes involved in carcinogen detoxification and downregulation of genes associated with cancer progression pathways. These molecular changes were correlated with improved clinical outcomes, supporting SFN’s potential as an adjunctive therapy.

Breast cancer studies have provided additional insights into SFN’s clinical activity. A foundational pilot study demonstrated that following oral administration of a broccoli sprout preparation, sulforaphane metabolites are delivered to and can be measured in human breast tissue, providing critical proof-of-concept for its direct bioactivity in the target organ (99). The intervention led to promoter hypermethylation of critical genes involved in Wnt signaling and inflammation, pathways fundamentally implicated in breast carcinogenesis. This epigenetic reprogramming suggests a potential mechanism for SFN’s protective effects in breast tissue.

In the context of melanoma prevention, clinical investigations have revealed SFN’s capacity to modulate ultraviolet radiation-induced damage. A randomized controlled trial demonstrated that topical application of SFN-rich extracts significantly reduced ultraviolet-induced erythema and DNA damage in human skin (86). The protective effects were associated with enhanced expression of phase II enzymes and reduced inflammatory responses, providing mechanistic insights into SFN’s photoprotective properties.

The accumulating clinical evidence consistently demonstrates SFN’s ability to modulate molecular pathways relevant to carcinogenesis across different tissue types. However, these studies also highlight important challenges in clinical translation, particularly regarding interindividual variability in response and the need for optimized delivery strategies. Future clinical development should focus on biomarker-guided patient selection and the development of formulations that ensure consistent bioavailability to maximize therapeutic efficacy.

### 5.3 Addressing translational challenges

Despite these promising results, several challenges must be addressed to optimize SFN’s clinical application. The substantial interindividual variability in SFN bioavailability, driven by differences in gut microbiota composition and GST polymorphisms, necessitates personalized dosing strategies (100, 101). Future clinical protocols should incorporate biomarker-guided approaches to identify optimal responders and tailor interventions accordingly.

The formulation and delivery of SFN present additional hurdles. Conventional oral administration faces limitations due to SFN’s rapid metabolism and variable bioavailability. Emerging nanotechnology approaches, including polymeric nanoparticles and lipid-based delivery systems, show promise in enhancing SFN stability, prolonging circulation time, and improving tumor-specific delivery (94, 102). These advanced formulations could potentially overcome the pharmacokinetic limitations that have historically constrained SFN’s clinical efficacy.

Furthermore, the optimal timing and sequencing of SFN administration in combination therapies require careful consideration. The dual nature of Nrf2 activation—protective in normal tissues but potentially protective of tumor cells under certain conditions—demands precise scheduling to maximize therapeutic synergy while minimizing potential interference with conventional treatments (6, 63). The accumulating clinical evidence, while still evolving, provides a solid foundation for the continued development of SFN as both a chemopreventive agent and therapeutic adjunct. Future research directions should focus on validating biomarkers of response, optimizing delivery systems, and conducting larger-scale trials in carefully selected patient populations to fully realize SFN’s potential in precision oncology.

## 6 Challenges and future perspectives

Despite the compelling preclinical evidence and promising early clinical results, the full translation of SFN’s potential into reliable clinical applications faces several significant challenges. This section outlines these barriers and proposes integrated strategies to overcome them, framing a future roadmap for SFN research and application.

### 6.1 Comprehensive strategies to overcome translational challenges

The clinical translation of SFN faces significant pharmacological hurdles that require a multidisciplinary approach. A primary challenge lies in SFN’s suboptimal pharmacokinetic profile, characterized by rapid metabolism, limited oral bioavailability, and chemical instability. To address these limitations, nanotechnology has emerged as a promising solution. Lipid-based nanoparticles, including solid lipid nanoparticles and nanoemulsions, have demonstrated enhanced protection of SFN from degradation in the gastrointestinal tract, while polymeric nanoparticles such as PLGA-based systems enable sustained release profiles that maintain therapeutic concentrations over extended periods. Surface functionalization of these nanocarriers with targeting ligands (e.g., folate, transferrin) further enhances their specificity, directing SFN to tumor tissues while minimizing systemic exposure (103). It is noteworthy that such delivery challenges are not unique to SFN but represent a common hurdle for many bioactive phytochemicals, as comprehensively documented in the case of curcumin where nano-formulations have successfully addressed similar bioavailability limitations. These advanced delivery systems have shown remarkable success in preclinical models, improving SFN’s antitumor efficacy by 3- to 5-fold compared to free compound administration (104).

Beyond technological innovations in drug delivery, addressing the substantial interindividual variability in SFN response is equally crucial. This variability, driven by host genetics and gut microbiome composition, necessitates personalized intervention strategies. Genetic polymorphisms in GSTs, particularly the GSTM1 and GSTT1 null genotypes, significantly influence SFN’s metabolic fate and clinical efficacy. The implementation of GST genotyping could identify optimal responders who would derive maximum benefit from SFN supplementation (105, 106). Concurrently, modulating the gut microbiome through specific probiotic supplements (e.g., Lactobacillus and Bifidobacterium strains with high myrosinase activity) represents a promising strategy to standardize and enhance the conversion of glucoraphanin to bioactive SFN, particularly when dietary SFN is obtained from cooked vegetables where plant myrosinase is inactivated (100, 101).

At the most fundamental level, enhancing the glucoraphanin content in cruciferous vegetables through genetic engineering provides a sustainable, scalable approach to SFN-based prevention. The elucidation of glucoraphanin’s biosynthetic pathway and its regulatory mechanisms, particularly the master transcription factor MYB28, has enabled targeted genetic interventions. CRISPR-Cas9-mediated genome editing has successfully generated Brassica varieties with significantly increased glucoraphanin accumulation by modulating key genes in the pathway, including MYB28, AOP2, and BCAT4 (9, 27, 96, 107). These biofortified crops not only offer a practical solution for population-level chemoprevention but also represent a cost-effective alternative to purified supplements, potentially increasing accessibility across diverse socioeconomic groups.

### 6.2 Concluding remarks and future directions

In conclusion, this review has systematically traced SFN’s journey from its biosynthetic origins in plants to its multifaceted mechanisms of action in human cancer prevention and therapy, ultimately addressing the translational challenges that currently limit its clinical application. Three key insights emerge from this comprehensive analysis.

First, SFN stands as a exemplary multi-targeted agent whose pleiotropic mechanisms—spanning epigenetic regulation, induction of specialized cell death programs, immunomodulation, and CSC targeting—provide a robust foundation for its efficacy against heterogeneous and treatment-resistant malignancies. Unlike many single-target agents, SFN’s ability to simultaneously engage multiple vulnerability nodes in cancer cells reduces the likelihood of resistance development and enhances its therapeutic potential.

Second, the successful clinical translation of SFN depends fundamentally on overcoming the substantial interindividual variability in its bioavailability and metabolism. Future research must prioritize the development of validated biomarkers for patient stratification and the implementation of precision nutrition approaches that account for genetic polymorphisms and microbiome variations. The establishment of predictive biomarkers will enable the identification of optimal responders and the customization of dosing regimens to maximize therapeutic outcomes.

Finally, the full realization of SFN’s potential will require the continued convergence of cutting-edge technologies from diverse fields. Nanotechnology-driven delivery systems, microbiome engineering, and CRISPR-based crop biofortification represent complementary strategies that collectively address the key limitations of current SFN formulations. The integration of these approaches will facilitate the transition from one-size-fits-all supplementation to targeted, effective, and sustainable interventions.

Looking forward, the future of SFN research lies in well-designed, biomarker-stratified clinical trials that incorporate advanced formulations and consider the complex interplay between diet, host genetics, and gut microbiota. By embracing this integrated, multidisciplinary approach, the scientific community can fully unlock the potential of this remarkable phytochemical, ultimately transforming SFN from a promising dietary compound into a reliable tool for cancer prevention and therapy.
